# Differentially expressed immune response genes in COVID-19 patients based on disease severity

**DOI:** 10.18632/aging.202877

**Published:** 2021-03-29

**Authors:** Shasha Li, Xiaoqiong Duan, Yujia Li, Ming Li, Yong Gao, Tuantuan Li, Shilin Li, Lin Tan, Tuo Shao, Andre J. Jeyarajan, Limin Chen, Mingfeng Han, Wenyu Lin, Xiuyong Li

**Affiliations:** 1 Department of Hepatology, The Second People's Hospital of Fuyang City, Fuyang 236015, Anhui Province, P.R. of China; 2 Fuyang Infectious Disease Clinical College of Anhui Medical University, Fuyang 236015, Anhui Province, P.R. of China; 3 Institute of Blood Transfusion, Chinese Academy of Medical Sciences and Peking Union Medical College, Chengdu 610052, Sichuan Province, P.R. of China; 4 Clinical laboratory, The Second People's Hospital of Fuyang City, Fuyang 236015, Anhui Province, P.R. of China; 5 Liver Center and Gastrointestinal Division, Department of Medicine, Massachusetts General Hospital, Harvard Medical School, Boston, MA 02114, USA; 6 Department of Pneumology, The Second People's Hospital of Fuyang City, Fuyang 236015, Anhui Province, P.R. of China; 7 Hemodialysis center, The Second People's Hospital of Fuyang City, Fuyang 236015, Anhui Province, P.R. of China

**Keywords:** COVID-19, SARS-CoV-2, immune response genes, IL6, APOBEC3G

## Abstract

Background: Dysregulated immune responses to severe acute respiratory syndrome coronavirus 2 (SARS-CoV-2) are thought to underlie the progression of coronavirus disease 2019 (COVID-19). We sought to further characterize host antiviral and cytokine gene expression in COVID-19 patients based on illness severity.

Methods: In this case-control study, we retrospectively analyzed 46 recovered COVID-19 patients and 24 healthy subjects (no history of COVID-19) recruited from the Second People's Hospital of Fuyang City. Blood samples were collected from each study participant for RNA extraction and PCR. We assessed changes in antiviral gene expression between healthy controls and patients with mild/moderate (MM) and severe/critical (SC) disease.

Results: We found that type I interferon signaling (IFNA2, TLR8, IFNA1, IFNAR1, TLR9, IRF7, ISG15, APOBEC3G, and MX1) and genes encoding proinflammatory cytokines (IL12B, IL15, IL6, IL12A and IL1B) and chemokines (CXCL9, CXCL11 and CXCL10) were upregulated in patients with MM and SC disease. Moreover, we found that IFNA1, apolipoprotein B mRNA editing enzyme, catalytic polypeptide-like 3G (APOBEC3G), and Fas-associated protein with death domain (FADD) were significantly downregulated (*P* < 0.05) in the SC group compared to the MM group. We also observed that microRNA (miR)-155 and miR-130a levels were markedly higher in the MM group compared to the SC group.

Conclusion: COVID-19 is associated with the activation of host antiviral genes. Induction of the IFN system appears to be particularly important in controlling SARS-CoV-2 infection, as decreased expression of IFNA1, APOBEC3G and FADD genes in SC patients, relative to MM patients, may be associated with disease progression.

## INTRODUCTION

Coronavirus disease 2019 (COVID-19) is an infectious disease caused by severe acute respiratory syndrome coronavirus 2 (SARS-CoV-2) [1–6]. Infection with SARS-CoV-2 is often asymptomatic but symptomatic cases most commonly present with fever, myalgia, fatigue, loss of taste or smell and upper respiratory symptoms including dry cough, dyspnea, and sore throat [1, 7]. COVID-19 has an extensive clinical course ranging from mild/moderate (MM) to severe/critical (SC) disease, in which the disease has progressed to pneumonia, acute respiratory distress syndrome, or multi-organ failure. Notably, in the largest cases series to date, approximately 10% of patients required intensive care admission [1, 6, 8]. SARS-CoV-2 has infected more than 88 million people worldwide, resulting in over 1.9 million deaths as of January 8, 2021 [9–12]. Due to its worldwide spread and severity, the World Health Organization has declared COVID-19 a pandemic [13–15]. Given no effective antiviral treatment and slow vaccine rollout, COVID-19 continues to be a serious threat to public health worldwide (see the Coronavirus Resource Centre or the latest worldwide data (https://coronavirus.jhu.edu/) [16].

As with other respiratory viruses, SARS-CoV-2 infects cells of the respiratory mucosa through the inhalation of viral particles or contact between contaminated surfaces and mucous membranes, such as those of the nose or eyes [7, 17–19]. Angiotensin converting enzyme 2 (ACE2) is one of the major entry receptors for SARS-CoV-2 [7, 20]. The host immune response to SARS-CoV-2 infection involves activation of both cellular and humoral arms. The innate immune system recognizes pathogens and induces production of proinflammatory cytokines and chemokines that trigger the adaptive immune system. This response involves activation of cytotoxic T lymphocytes (CTLs) which directly kill virus-infected cells and B cell differentiation into plasma cells that produce pathogen-specific antibodies in the serum and at mucosal surfaces [7]. SARS-CoV-2 infection in mucosal epithelial cells and macrophages activates several innate immune response pathways which are mediated by pattern recognition receptors (PRRs) including toll-like receptors (TLRs), NOD-like receptors (NLRs), and cytoplasmic DNA sensors such as RIG-I MAVS. These receptors recognize and bind viral DNA and RNA, activating downstream signaling pathways to induce the production of inflammatory cytokines including interferons (IFNs). IFN α and β mediate type-I interferon signaling that activates dendritic and natural killer cells as well as the adaptive immune response [21–24]. Activation of the IFN system subsequently stimulates antiviral gene expression in neighboring cells and recruits other immune cells involved in tissue repair and homeostasis.

Dysregulation of the innate immune response contributes to cytokine storm observed in severe COVID-19 patients [7, 19]. It has been reported that inborn errors of TLR3 and IRF7 dependent type-I IFN immunity may be associated with COVID-19 symptoms and outcomes [25, 26]. However, the molecular mechanisms that determine why some individuals suffer from severe illness whilst others are asymptomatic or exhibit mild/moderate symptoms are not well understood. Gene expression profiling technologies including PCR arrays and microarrays enable one to evaluate gene expression patterns on a specific or genome-wide scale [7, 19]. Human Antiviral Response PCR arrays contain receptors and signaling effectors for TLRs, NLRs and RIG-I-like receptors, as well as the genes regulated by these pathways and thus can be utilized to analyze type-I interferon signaling and downstream interferon-stimulated gene (ISG) expression in COVID-19 patients. We recently reported that COVID-19-induced liver dysfunction is associated with increasing age in 159 patients in a Chinese Fuyang City hospital [6]. In the present case-control study, we used PCR array and quantitative real time PCR (qPCR) to analyze the expression of a panel of genes that may be implicated in regulating COVID-19 progression in a subset of recovered patients from this cohort at the Second People's Hospital of Fuyang City in China. We sought to explore the relationship between host immune response gene expression and symptoms experienced by COVID-19 patients.

## RESULTS

### Patient demographic and baseline characteristics

We recruited 46 recovered COVID-19 patients from the Second People's Hospital of Fuyang City. Of these patients, 30 had MM disease and 16 had SC disease, as in our previous report [6]. Demographic and clinical characteristics of the 46 recovered COVID-19 and 24 negative control patients are provided in Table 1.

**Table 1:** Comparison of demographics and clinical characteristics between SC, MM and control patients.

**Characteristic**	**SC**	**MM**	**Control**	**P value**	**P1 value**	**P2 value**	**P3 value**
Total number (n)	16	30	24				
Age (years) (median, IQR)	54.0(49.3-65.5)	48.0(37.0-59.3)	36.0(25.8-54.3)	0.002	0.110	0.001	0.189
Male gender (n, %)	13(81.3%)	16(53.3%)	10(41.7%)	0.045			
Body mass index (kg/m2) (Median, IQR)	26.2(24.5-28.5)	24.5(23.1-26.2)	23.1(19.2-25.9)	0.049	0.763	0.05	0.261
Temperature (° C) (Median, IQR)	36.3(36.1-36.7)	36.2(36.1-36.5)	36.2(36.1-36.4)	0.712			
Heart rate(beats / minute) (Median, IQR)	72(65-80)	77(68-86)	69(68-76)	0.335			
Blood Pressure (mmHg) (Median, IQR)	140(113-150)/	127(118-139)/	122(117-136)/77(65-84)	0.522/0.002	0.522/0.016	0.522/0.003	0.522/1.000
	93(84-100)	90(82-95)					
Respiratory rate (breaths / minute) (Median, IQR)	18(17-19)	18(17-19)	17(16-19)	0.712			
WBC	5.2(4.5-5.8)	5.4(4.5-5.9	6.7(5.4-7.5)	0.008	0.041	0.011	1
RBC	4.3(4.0-4.7))	4.4(3.8-4.7)	4.5(4.1-4.8)	0.446			
Oxygen saturation (%) (Median, IQR)	98(97-98)	98(97-99)	99(98-99)	0.002	0.020	0.003	0.973
**Comorbidities**	**SC**	**MM**	**Control**				
Chronic hepatitis B virus (HBV)	2	3	1				
Hypertension	6	6	1				
Diabetes	2	0	1				
Fatty liver	1	1	3				
Other	4	5	3				

P1, P2, and P3 represent the statistical significance values calculated using T-tests for comparisons between Control-MM, Control-SC, and MM-SC groups, respectively. Values are expressed as median (range, 25-75%). Data were analyzed using SPSS Statistics v25.0 (Armonk, New York, USA). Continuous data are expressed as medians with interquartile range, and categorical data as frequencies. Groups were compared using analysis of variance and non-parametric tests, and correlations between clinical and laboratory parameters were evaluated using the two-tailed chi-squared test.

### Host antiviral response genes differentially expressed in COVID-19 patients

**Figure 1:**
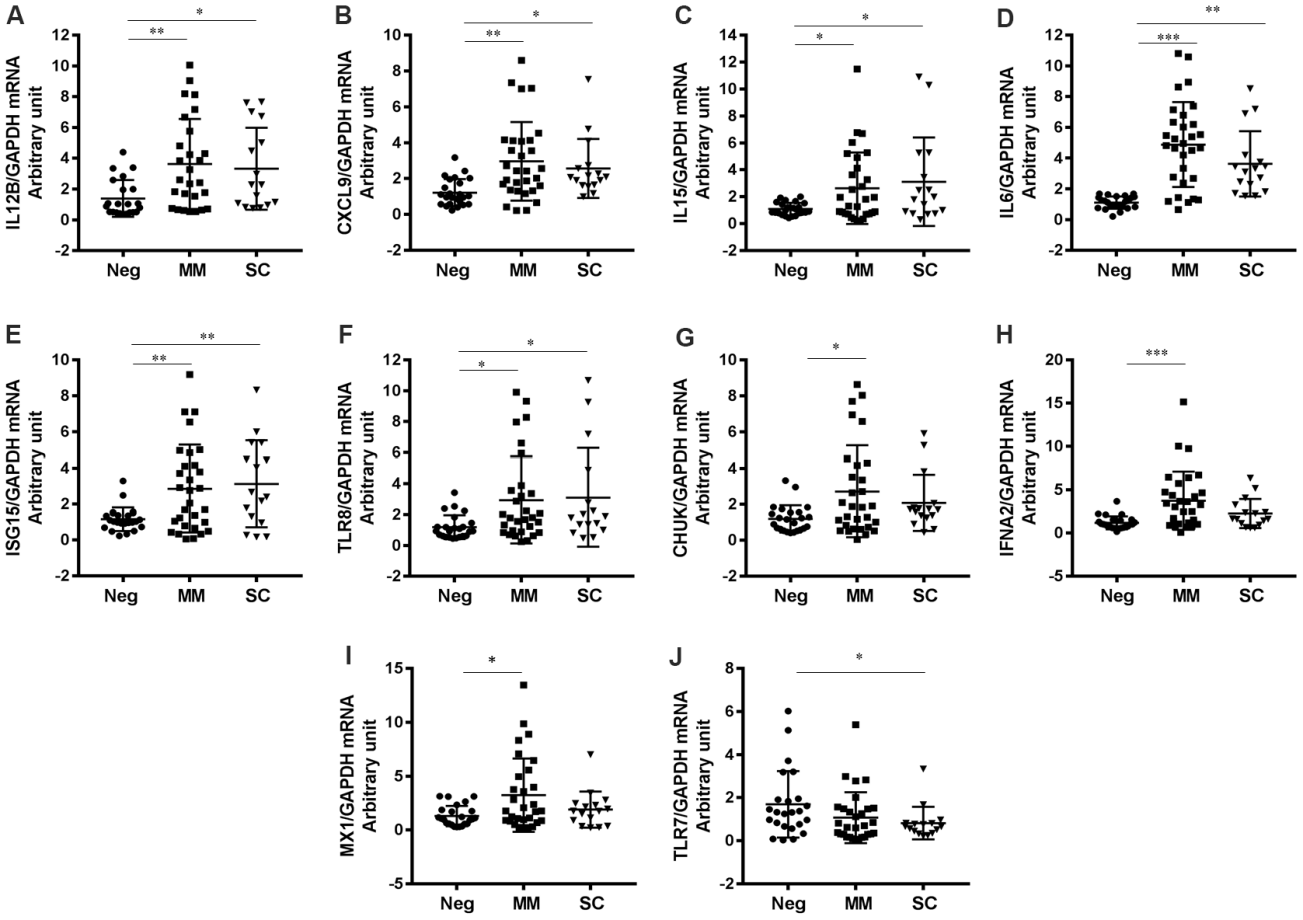
**Genes differentially expressed in COVID-19 patients based on disease severity.** (**A**) IL12B (P=0.0054 for MM vs Neg; P=0.0445 for SC vs Neg), (**B**) CXCL9 (P=0.0010 for MM vs Neg; P=0.0428 for SC vs Neg), (**C**) IL15 (P=0.0500 for MM vs Neg; P=0.0262 for SC vs Neg), (**D**) IL6 (P=0.0009 for MM vs Neg; P=0.0011 for SC vs Neg), (**E**) ISG15, (**F**) TLR8 (P=0.0278 for MM vs Neg; P=0.0440 for SC vs Neg), (**G**) CHUK (P=0.0121 for MM vs Neg), (**H**) IFNA2 (P=0.0006 for MM vs Neg), (**I**) MX1 (P=0.0134 for MM vs Neg), (**J**) TLR7 (P=0.0303 for SC vs Neg). *P<0.05, **P<0.01, ***P<0.001.

To investigate the antiviral genes differentially expressed in COVID-19 patients, particularly between patients with MM and SC disease, we performed a human antiviral response RT^2^ Profilier^TM^ PCR array (PAHS-122ZA). We monitored 84 human immunity-related genes in this PCR array and found that most genes were expressed at a higher-level in COVID-19 patients than in healthy negative controls. We identified 20 genes in the MM group and 12 genes in the SC group (10 overlapping genes) that were elevated by at least 2-fold compared to healthy subjects (Table 2). We performed qPCR to further validate the differentially expressed genes in MM, SC and negative control patients. Notably, the gene expression measurements obtained by qPCR were consistent with those derived by PCR array (Figure 1, Table 2). The upregulated genes were primarily involved in activation of the IFN system (IFNA2, TLR8, IFNA1, IFNAR1, TLR9, IRF7, ISG15, APOBEC3G and MX1) and encoded for proinflammatory cytokines (IL12B, IL15 and IL6) and chemokines (CXCL9, CXCL11 and CXCL10). In particular, we found that IRF7 and TLR3 were significantly induced in MM patients compared to the negative controls (Table 2). Interestingly, we did not observe marked decreases in gene expression between the MM and negative control group.

**Table 2:** List of selected differentially expressed antiviral genes identified by PCR array.

**Gene name**	**Fold change**	
	**MM/Neg**	**SC/Neg**
IL12B	11.42**	11.15*
IFNA2	6.59*	2.57*
CXCL9	6.52*	3.89*
TLR8	5.42*	4.80
IL15	5.30*	4.58*
IFNA1	5.22**	1.92*
IFNAR1	4.75	3.77*
CXCL11	4.55*	2.25
CHUK	4.53*	2.81
IL6	3.64*	3.13*
TLR9	3.31*	1.44
IL12A	2.94**	2.79*
SPP1	2.81	1.51
IRF7	2.77**	1.62
CASP1	2.48	1.32
ISG15	2.36	0.89
APOBEC3G	2.21	0.78
CXCL10	2.18**	1.47
MX1	2.16	0.62
PYDC1	2.06*	1.98
CTSL	1.92	1.86
NLRP3	1.90	1.28
TLR3	1.87*	1.54
CD80	1.80*	1.30
AZ12	1.80	1.58
NFKBIA	1.78*	2.34
MAPK8	1.75	1.89*
PSTPIP1	1.69**	1.41
JUN	1.61	1.39
CXCL8	1.59	1.28
TICAM1	1.59	0.99
TRAF3	1.54	1.75
CYLD	1.49	1.25
CASP8	1.48	1.54
STAT1	1.44	0.87
MAP3K1	1.43	1.48
CCL3	1.38	1.59
HSP90AA1	1.37	0.93
PYCARD	1.33	0.74
IFNB1	1.32	1.04
MYD88	1.28	0.81
CASP10	1.27	1.63
DDX3X	1.26	1.46
NFKB1	1.23	0.94
TBK1	1.22	0.89
IL18	1.21	0.99
RELA	1.21	1.62
MAP2K1	1.20	1.01
TNF	1.19	1.06
CCL5	1.17	1.23
FADD	1.15	0.36*
IRF5	1.14	0.82
MAPK3	1.12	1.13
IRF3	1.12	1.05
ATG5	1.12	1.55
MAP2K3	1.12	1.06
IKBKB	1.10	1.49
OAS2	1.08	0.77
NOD2	1.06	1.69
CD86	1.05	0.67
MAVS	1.05	0.89
SUGT1	1.04	1.54
DHX58	1.03	0.59
CD40	0.98	0.68
TRAF6	0.98	1.12
AIM2	0.97	0.73
DDX58	0.96	1.34
MAP3K7	0.94	0.98
CTSS	0.93	0.67
RIPK1	0.91	0.74
CARD9	0.91	0.99
TRADD	0.87	0.53
IRAK1	0.84	0.76
IFIH1	0.84	0.52
PIN1	0.76	0.58
MAPK14	0.75	0.77
TRIM25	0.74	1.06
FOS	0.72	0.70
CTSB	0.72	0.85
MAPK1	0.66	0.73
TLR7	0.65	0.20
TKFC	0.64	0.63
IL1B	0.54	0.91
MEFV	0.50	0.62

Samples from three randomly selected study subjects were included in each group (MM, SC or negative) in the PCR array, Fold change was calculated relative to the negative control group. *P <0.05; **P <0.01.

**Figure 2:**
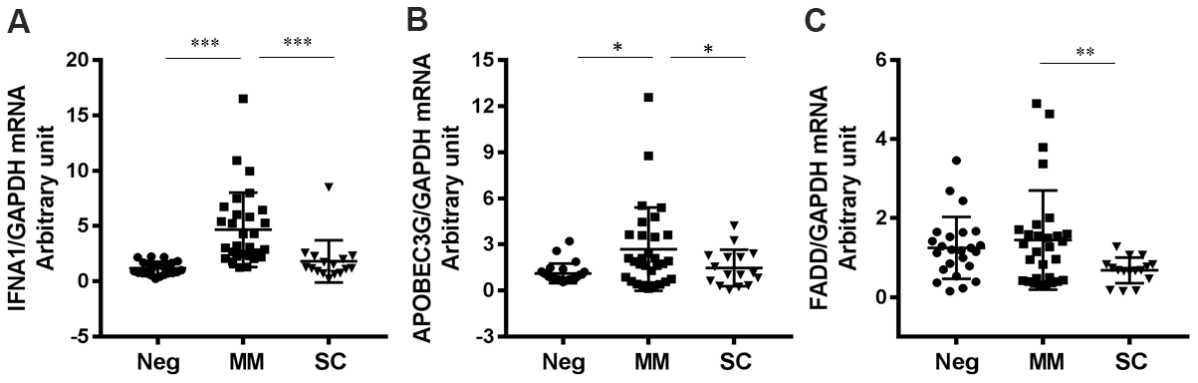
**Antiviral genes downregulated in SC patients compared to MM patients.** (**A**) IFNA1 (P<0.0001 for MM vs Neg; P=0.0007 for SC vs MM), (**B**) APOBEC3G (P=0.0100 for MM vs Neg; P=0.0408 for SC vs MM) and (**C**) FADD (P=0.0054 for MM vs Neg; P=0.0031 for SC vs MM). *P<0.05, **P<0.01, ***P<0.001.

However, the expression levels of two genes (FADD and TLR7) were significantly decreased in SC patients relative to MM patients (Figures 1, 2).

### Interferon-stimulated genes were differentially expressed in patients with mild and severe disease

We found that expression levels of interferon alpha 1 (IFNA1), apolipoprotein B mRNA editing enzyme, catalytic polypeptide-like 3G (APOBEC3G), and Fas-associated protein with death domain (FADD) were significantly lower (*P <* 0.05) in the SC group than in the MM group (Figure 2). Notably, IFNA1 are APOBEC3G are interferon-stimulated genes (ISGs); IFNA1 encodes IFNα, which plays a critical role in antiviral activities and APOBEC3G (part of the APOBEC superfamily of proteins) is believed to function in innate antiviral immunity [27]. In addition, we found that two additional IFN pathway genes, IFNA2 and MX1, were slightly downregulated in the SC group compared to the MM group. These results suggest that the IFN system is important in regulating COVID-19 progression. FADD is a key adaptor molecule involved in numerous physiological processes including mediating the extrinsic apoptotic pathway and regulating type I interferon signaling [28]. Of note, it has previously been reported that FADD was significantly reduced in HBV-infected patients [29].

### Antiviral genes not significantly upregulated in patients with severe/critical COVID-19

**Figure 3:**
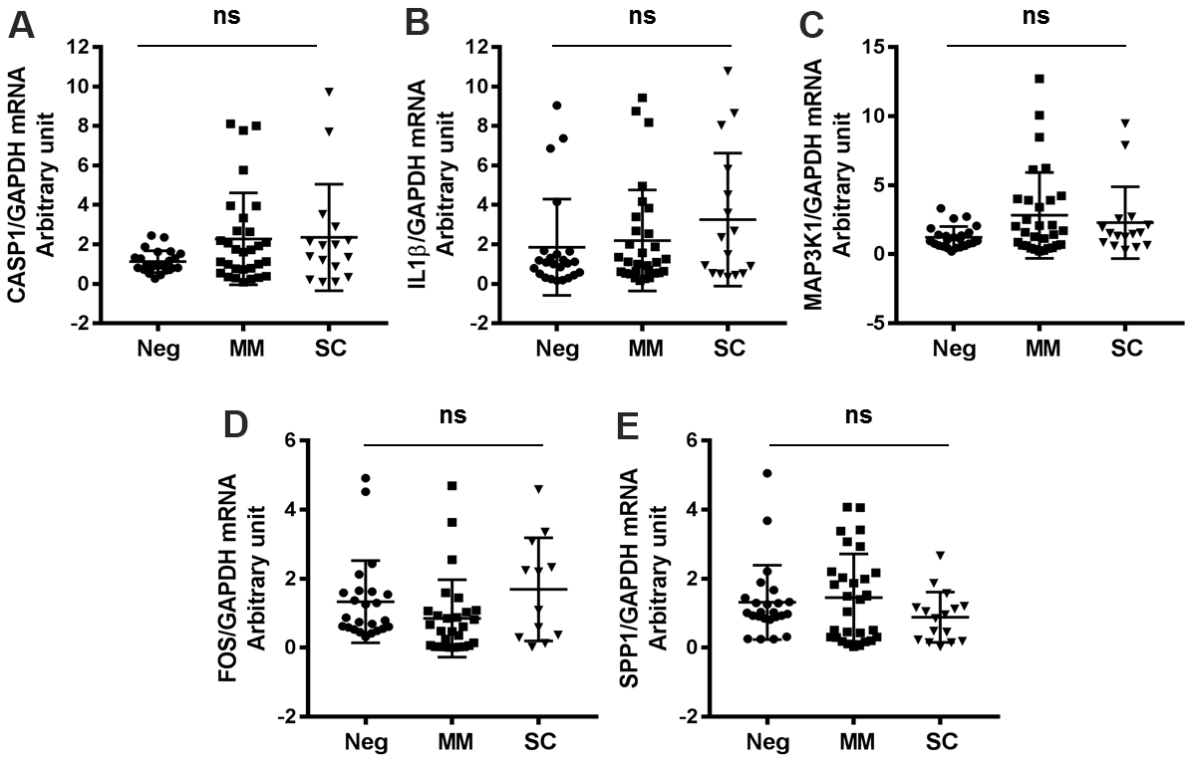
**Antiviral genes not significantly changed in COVID-19 patients and healthy controls.** (**A**) CASP1, (**B**) IL1β, (**C**) MAP3K1, (**D**) FOS, (**E**) SPP1.

We identified several antiviral genes that were not significantly activated in COVID-19 patients. Specifically, CASP1, IL1β, MAP3K1, FOS, and SPP1 mRNA expression levels were not markedly different between MM, SC and negative control patients (Figure 3, Table 2). These findings suggest that SARS-CoV-2 infection does not broadly activate host antiviral gene expression.

### Expression levels of other genes implicated in SARS-CoV-2 infection and COVID-19 progression

**Figure 4:**
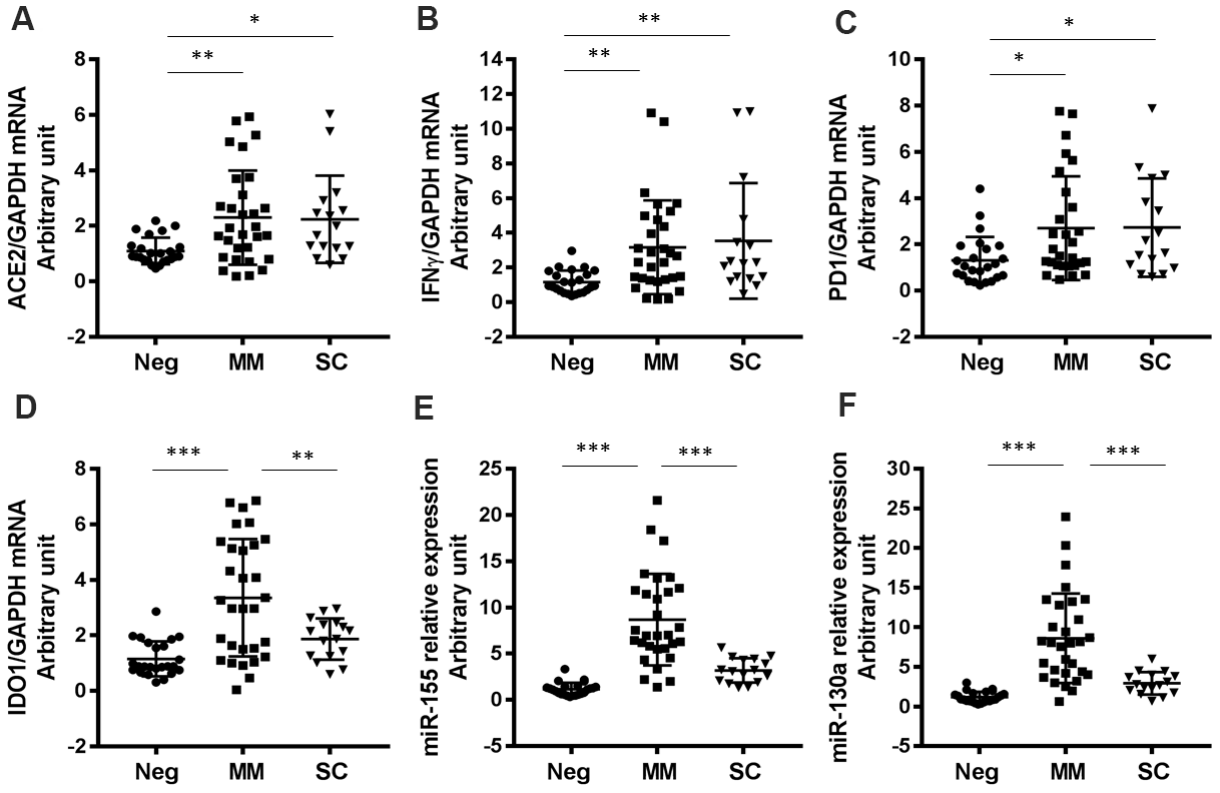
**Expression levels of other COVID-19-related genes in patients with varying degrees of disease severity.** (**A**) ACE2 (P=0.0057 for MM vs Neg; P=0.0313 for SC vs Neg), (**B**) IFNγ (P=0.0095 for MM vs Neg; P=0.0091 for SC vs Neg), (**C**) PD1 (P=0.0248 for MM vs Neg; P=0.0353 for SC vs Neg), (**D**) IDO1 (P<0.0001 for MM vs Neg; P=0.0052 for SC vs MM), (**E**) miR-155 (P<0.0001 for MM vs Neg; P<0.0001 for SC vs MM), (**F**) miR-130a (P<0.0001 for MM vs Neg; P<0.0001 for SC vs MM). *P<0.05, **P<0.01, ***P<0.001.

We also evaluated genes that have been reported to regulate SARS-CoV-2 infection or host antiviral immunity. For example, expression of angiotensin-converting enzyme 2 (ACE2), which is the entry receptor for SARS-CoV-2, was substantially higher in both MM and SC groups than in the negative control group (Figure 4A). Furthermore, we found that IFNγ and PD-1 expression levels were significantly increased in all SARS-CoV-2-infected patients compared to negative controls (Figure 4B, 4C). Indoleamine 2,3-dioxygenase (IDO1) levels were higher in the MM group than in the control and severe groups (Figure 4D). The microRNAs, miR-155 and miR-130a have been reported to regulate viral infection by regulating the host immune response [30–32]. In this study, we found that both miR-155 and miR-130a were significantly upregulated in the MM disease group compared to the SC group (Figure 4E, 4F).

## DISCUSSION

ISGs are thought to be the primary effectors against many viral infections including human coronaviruses (HCoVs), hepatitis C virus (HCV) and HBV infections [7, 19, 23, 24, 26, 33, 34]. The host IFN signaling pathway and baseline ISG levels have been reported to be associated with treatment outcomes in HCV-infected patients [35]. TLR3 and IRF7 are vital mediators of type I IFN-induced immunity against SARS-CoV-2 infection [26]. Our findings illustrating increased IRF7 and IRF3 mRNA levels in COVID-19 patients with mild/moderate disease further confirm their role in the host antiviral response. APOBEC3 proteins bind to the coronavirus nucleoprotein that restricts HCoVs RNA replication and notably, APOBEC3-family gene expression is upregulated in HCoVs-infected cells [33]. Likewise, we observed significantly increased APOBEC3G mRNA levels in MM patients compared to SC patients. Our findings suggest that decreased APOBEC3G expression may serve as a biomarker for severe illness in SARS-CoV-2-infected patients.

Host cytokine perturbations have been observed in COVID-19 patients, particularly in SARS-CoV-2-induced cytokine storm syndrome (CSS), which is associated with disease severity [6, 7, 26]. The overactive immune response and nonspecific, disseminated cytokine release, including interleukin-6 (IL-6), in severe cases of COVID-19 may lead to organ failure [36]. Moreover, the combination of several cytokine levels has been used to predict COVID-19 severity and recovery [37]. IL-6 (proinflammatory) and IL-10 (anti-inflammatory) are two cytokines commonly dysregulated in COVID-19 patients and the ratio of IL6-to-IL10 may be used to predict outcomes of COVID-19 patients [37–39]. This new scoring system has been reported to help clinicians predict COVID-19 patients at risk for severe disease and those that require intervention [38]. We found that IL6 mRNA levels were significantly elevated in both MM and SC patients compared to negative controls. Our findings suggest that SARS-CoV-2 induces proinflammatory gene expression.

It has previously been reported that SARS-CoV-2 infection activates the host IFN system and upregulates expression of ACE2 (a critical entry receptor for SARS-CoV-2) [40]. We similarly found that expression of ACE2 was significantly induced in both MM and SC patient groups compared to negative controls. These findings suggest that SARS-CoV-2 infection induces ACE2 expression or that patients with elevated baseline ACE2 expression are more susceptible to severe COVID-19 illness. MicroRNAs have been reported to play an important role in host antiviral immunity. Notably, miR-155 is a multifunctional microRNA that has an indispensable capacity in immune responses [41]. We previously reported that miR-130a regulates HCV and HBV infection through the IFN pathway [32, 42]. In the present study, we also found that miR-155 and miR-130a were significantly upregulated in COVID-19 patients when compared to negative controls. It is possible that SARS-CoV-2-mediated regulation of ISGs may occur partially through miRNAs such as miR-155 and miR-130a. We previously reported that COVID-19-induced liver function abnormalities were relatively mild/moderate in this cohort [6]. This study also highlighted the importance of careful liver function management in these patients, particularly among older patients and those with underlying liver disease [6].

In summary, our study adds to the growing body of evidence that SARS-CoV-2 infection, recovery and disease severity are associated with deviations in host interferon responses. Specifically, we found that the expression of genes encoding proinflammatory cytokines (IL12B, IL15, IL6, IL12A and IL1B) and chemokines (CXCL9, CXCL11 and CXCL10) was broadly increased in COVID-19 patients and speculate that other viral infections may also induce expression of these genes. Activation of ISGs appear to be beneficial in controlling SARS-CoV-2, but over-activation of proinflammatory cytokines and downregulation of certain ISGs (IFNA1, APOBEC3G, and FADD) and microRNAs (miR-155 and miR-130a) may be associated with progression to severe/critical COVID-19. The present study has several limitations including a relatively small sample size and lack of blood samples from patients with current SARS-CoV-2 infection in Fuyang City, China. Furthermore, the age of the control group is significantly lower than that of the studied group. A more detailed understanding of the interactions between host ISGs, cytokines and SARS-CoV-2, as well as viral pathogenesis and antiviral responses in COVID-19 are therefore required in order to better predict disease severity and to optimize patient management.

## MATERIALS AND METHODS

### Ethics approval and consent to participate

This study was registered at the Chinese Clinical Trial Registry (registration number: ChiCTR2000031620). The study protocol was approved by the Ethics Review Committee of the Second People's Hospital of Fuyang City (reference number: 2020006). This research was conducted in accordance with the ethical standards of the institutional and national research committees, and with the 1964 declaration of Helsinki.

### Patients and study design

The Second People's Hospital of Fuyang City had originally admitted 159 patients with confirmed SARS-CoV-2 infection during the outbreak of the disease in Anhui Provence from January to March 2020 [6]. There were no SARS-CoV-2-related deaths in this hospital. COVID-19 diagnosis, laboratory testing, and treatment in this cohort have been previously described [6]. Patients with confirmed COVID-19 were classified as having had mild/moderate (MM) or severe/critical (SC) disease based on symptomatology [6]. We recruited 46 recovered COVID-19 patients (post-hospital discharge) from this cohort and 24 healthy subjects, age and gender matched, with no history of SARS-CoV-2 infection (negative control) from the Physical Examination Center in the Second People's Hospital of Fuyang City (Table 1). Blood samples were collected from each study subject for RNA extraction and PCR. We sought to assess changes in antiviral gene expression between patients with MM and SC disease and healthy controls.

### RNA isolation

Total RNA from patient whole blood was extracted using TRIzol reagent (Invitrogen, CA, USA) as previously described [42].

### RT^2^ Profiler™ PCR array

The RT^2^ Profiler™ PCR array (PAHS-122ZA) for human antiviral responses was purchased from Qiagen (Qiagen, MD, USA). The first strand cDNA was synthesized using ReverTra Ace® qPCR RT Master Mix with gDNA remover (Toyobo, Osaka, Japan). The cDNA was diluted with nuclease-free water and mixed with NovoStar SYBR qPCR SuperMix Plus with low ROX Premixed (Novoprotein, Shanghai, China) as previously described [32, 42]. Three samples from each group (SC, MM or negative control) were randomly selected and loaded into the 96-well RT^2^ Profiler™ PCR Array plate.

### Quantitative real time PCR and gene quantification

Selected gene mRNA expression levels for all samples were quantified by qPCR using the ABI QuantStudio 3 system (Applied Biosystems Inc, MA, USA). Relative mRNA expression levels for each gene were calculated using the ∆∆Ct method and normalized to GAPDH to obtain an arbitrary unit as previously described [32, 42]. The qRT-PCR primer sequences are listed in Table 3.

**Table 3:** List of primer sequences used for qPCR.

**Gene name**	**Forward primer**	**Reverse primer**
CXCL9	CCAGTAGTGAGAAAGGGTCGC	AGGGCTTGGGGCAAATTGTT
IL12B	ACCCTGACCATCCAAGTCAAA	TTGGCCTCGCATCTTAGAAAG
IFNA1	GCCTCGCCCTTTGCTTTACT	CTGTGGGTCTCAGGGAGATCA
IFNA2	GCTTGGGATGAGACCCTCCTA	CCCACCCCCTGTATCACAC
IL15	TTTCAGTGCAGGGCTTCCTAA	GGGTGAACATCACTTTCCGTAT
IFNAR1	AACAGGAGCGATGAGTCTGTC	TGCGAAATGGTGTAAATGAGTCA
CHUK	GGCTTCGGGAACGTCTGTC	TTTGGTACTTAGCTCTAGGCGA
IL6	CAGCCCTGAGAAAGGAGACAT	GGTTCAGGTTGTTTTCTGCCA
MX1	GTTTCCGAAGTGGACATCGCA	GAAGGGCAACTCCTGACAGT
TLR7	TCCTTGGGGCTAGATGGTTTC	TCCACGATCACATGGTTCTTTG
FADD	CCGCGCCTGGGGAAGAAG	CCAGCCTTCTCCAATCTTTCC
FOS	CCGGGGATAGCCTCTCTTACT	CCAGGTCCGTGCAGAAGTC
GAPDH	ACCTTCCCCATGGTGTCTGA	GCTCCTCCTGTTCGACAGTCA
SPP1	TGGGAATAGCTTTGGGAAGTGG	CCGATGTCCAAAGGTGCAAT
TLR8	ATGTTCCTTCAGTCGTCAATGC	TTGCTGCACTCTGCAATAACT
APOBEC3G	GCATCGTGACCAGGAGTATGA	GTCAGGGTAACCTTCGGGT
CASP1	CAGCCCTGGTGTGGTGTG	AAAATCCTTCTCTATGTGGGCTTTC
MAP3K1	TGATGTATGGAGTGTTGGCTG	AATGTGAAGGGATCGATGGAG
ACE2	AACTGCTGCTCAGTCCACC	AAAAGGCAGACCATTTGTCCC
IDO1	GCCAGCTTCGAGAAAGAGTTG	ATCCCAGAACTAGACGTGCAA

### Statistical analyses

Data analyses were performed using one-way ANOVA. Comparisons between negative control, MM and SC groups were analyzed by two-tailed T-tests as previously reported [6, 32, 42]. Data are expressed as the mean ± standard deviation of at least three biologic replicates, unless stated otherwise. Continuous data were expressed as medians with interquartile range, and categorical data as frequencies. Groups were compared using the Non-parametric test, and the correlations between clinical and laboratory parameters were evaluated using the two-tailed chi-squared test. In all analyses, * represents *P* < 0.05, ** represents *P* < 0.01 and *** represents *P* < 0.001 for indicated comparisons.
